# Comparing absorbed doses and radiation risk of the α-emitting bone-seekers [^223^Ra]RaCl_2_ and [^224^Ra]RaCl_2_

**DOI:** 10.3389/fmed.2022.1057373

**Published:** 2023-01-04

**Authors:** Michael Lassmann, Uta Eberlein

**Affiliations:** Department of Nuclear Medicine, University of Würzburg, Würzburg, Germany

**Keywords:** dosimetry, biodosimetry, ^224^Ra, ^223^Ra, epidemiology

## Abstract

[^223^Ra]RaCl_2_ and [^224^Ra]RaCl_2_ are bone seekers, emitting high LET, and short range (< 100 μm) alpha-particles. Both radionuclides show similar decay properties; the total alpha energies are comparable (^223^Ra: ≈28 MeV, ^224^Ra: ≈26 MeV). [^224^Ra]RaCl_2_ has been used from the mid-1940s until 1990 for treating different bone and joint diseases with activities of up to approximately 50 MBq [^224^Ra]RaCl_2_. In 2013 [^223^Ra]RaCl_2_ obtained marketing authorization by the FDA and by the European Union for the treatment of metastatic prostate cancer with an activity to administer of 0.055 MBq per kg body weight for six cycles. For intravenous injections in humans a model calculation using the biokinetic model of ICRP67 shows a ratio of organ absorbed dose coefficients (^224^Ra:^223^Ra) between 0.37 (liver) and 0.97 except for the kidneys (2.27) and blood (1.57). For the red marrow as primary organ-at-risk, the ratio is 0.57. The differences are mainly caused be the differing half-lives of the decay products of both radium isotopes. Both radionuclides show comparable DNA damage patterns in peripheral blood mononuclear cells after internal *ex-vivo* irradiation. Data on the long-term radiation-associated side effects are only available for treatment with [^224^Ra]RaCl_2_. Two epidemiological studies followed two patient groups treated with [^224^Ra]RaCl_2_ for more than 25 years. One of them was the “Spiess study”, a cohort of 899 juvenile patients who received several injections of [^224^Ra]RaCl_2_ with a mean specific activity of 0.66 MBq/kg. Another patient group of ankylosing spondylitis patients was treated with 10 repeated intravenous injections of [^224^Ra]RaCl_2_, 1 MBq each, 1 week apart. In total 1,471 of these patients were followed-up in the “Wick study”. In both studies, an increased cancer mortality by leukemia and solid cancers was observed. Similar considerations on long-term effects likely apply to [^223^Ra]RaCl_2_ as well since the biokinetics are similar and the absorbed doses in the same range. However, this increased risk will most likely not be observed due to the much shorter life expectancy of prostate cancer patients treated with [^223^Ra]RaCl_2_.

## Introduction

[^223^Ra]RaCl_2_ targets bone metastases with high LET and short range (< 100 μm) alpha-particles. In 2013, Parker et al. published the results of the phase III, double-blind, randomized, international ALSYMPCA study which compared [^223^Ra]RaCl_2_ plus best standard of care (BSC) vs. placebo plus BSC in castration resistant prostate cancer (CRPC) patients with bone metastases (1). The authors concluded that the ALSYMPCA study demonstrated significantly improved overall survival and low toxicity, suggesting that [^223^Ra]RaCl_2_ may provide a new standard of care for patients with CRPC and bone metastases. The results of the ALSYMPCA trial were used to obtain marketing authorization for [^223^Ra]RaCl_2_ (“XOFIGO”^®^) in Europe and North America in 2013.

[^224^Ra]RaCl_2_ has been used from the mid-1940s until 1990 for treating different bone and joint diseases, mainly in Germany (2, 3). After World War II, [^224^Ra]RaCl_2_ was primarily used for the treatment of children and juveniles suffering from bone tuberculosis, and even for the therapy of Ankylosing Spondylitis (AS) patients. The activities of [^224^Ra]RaCl_2_ administered at that time were high (approximately 0.66 MBq/kg body weight, corresponding to an activity of 50 MBq), with treatment durations ranging from 1 month to 45 months (median: 4 months). In the “Spiess study” 899 patients who received multiple injections of [^224^Ra]RaCl_2_ mainly between 1945 and 1955 for the treatment of tuberculosis, AS and some other diseases had been followed (3).

In a second group of patients who were treated with repeated intravenous injections of [^224^Ra]RaCl_2_ (excluding radiation therapy with X-rays) between 1948 and 1975 an epidemiological study on 1,471 ankylosing spondylitis patients was performed (“Wick study”). The activity was administered as 10 intravenous (IV) injections, 1 MBq each, one a week apart (mean: 0.17 MBq/kg, 10 MBq total). These patients have been followed together with a control group of 1,324 AS patients treated neither with radioactive drugs nor with X-rays (2).

[^224^Ra]RaCl_2_ has again been made available in Germany between 2000 and 2005 for treating AS. During that period, the German “Bundesinstitut für Arzneimittel und Medizinprodukte (BfArM)” approved an intraveneous injection of [^224^Ra]RaCl_2_ with total activities of 10 MBq (10 injections per week, 1 MBq each) for AS therapy (4).

[^224^Ra]RaCl_2_ has only been used in a small patient cohort for the treatment of osteoblastic metastases (5). Groth et al. describe the successful compassionate use treatment of osteoblastic metastases in 10 patients using 12 MBq or 20/30 MBq [^224^Ra]RaCl_2_. Except these studies, no further publications on patient treatment with [^224^Ra]RaCl_2_ are available.

The purpose of this work is to compare the dosimetry- and radiation-risk related aspects of treatments with [^223^Ra]RaCl_2_ and [^224^Ra]RaCl_2_.

## Radioactive decay and exposure

### Decay chains

#### ^223^Ra

**Figure 1:**
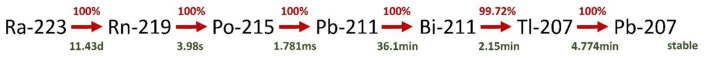
Decay chain of ^223^Ra. Decay products with branching ratios < 1% are omitted. The decay data were taken from http://www.nucleide.org/Laraweb/index.php.

^223^Ra is an alpha emitter (half-life = 11.43 d), which decays through a cascade of short-lived alpha- and beta-emitting progeny with the emission of about 20 MeV of energy per starting atom and the first two daughters and about 28 MeV through complete decay of the progeny to stable lead (Figure 1). A listing of the decay chain, branching ratios, half-lives, energies emitted by alpha-, beta, and gamma-transitions is provided e.g., by Schumann et al. (6). The data for the energy per transition in this publication was taken from the MIRD tables by Eckerman and Endo (7).

#### ^224^Ra

**Figure 2:**
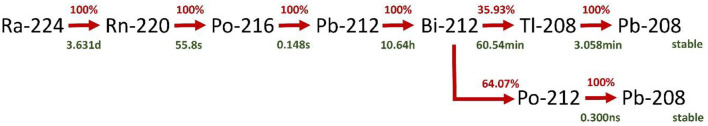
Decay chain of ^224^Ra. Decay products with branching ratios < 1% are omitted. The decay data were taken from http://www.nucleide.org/Laraweb/index.php.

^224^Ra is also an alpha emitter (half-life = 3.63 d) decaying through a cascade of short-lived alpha- and beta-emitting progeny with the emission of about 19 MeV of energy per starting atom and the first two daughters and about 26 MeV through complete decay of the progeny to stable lead (Figure 2). More details on the decay chain and the energies emitted are provided by Schumann et al. (6) and were also taken from the Eckerman and Endo tables (7).

## Biokinetics and dosimetry

### [^224^Ra]RaCl_2_

In 2002, Lassmann et al. (8) analyzed the dosimetry after the treatment of ankylosing spondylitis with [^224^Ra]RaCl_2_ by using model calculations based on ICRP 67 (9). Details on the model are provided in the publication by Lassmann et al. (8). The highest absorbed dose coefficients were found for bone endosteum (443 mGy/MBq), liver (14 mGy/MBq), and red bone marrow (44 mGy/MBq) (8).

### [^223^Ra]RaCl_2_

For [^223^Ra]RaCl_2_, Lassmann and Nosske (10) provided a first comprehensive model-based dosimetric calculation of organ doses after intravenous administration of [^223^Ra]RaCl_2_, in analogy to the previous publication by Lassmann et al. for [^224^Ra]RaCl_2_ (8). The highest absorbed dose coefficients were also found for bone endosteum (760 mGy/MBq), liver (38 mGy/MBq), and red bone marrow (78 mGy/MBq) (10).

Several clinical studies measured the disappearance of [^223^Ra]RaCl_2_ from the blood and the excretion pathways (11–14). All studies showed a rapid blood clearance; the major excretion pathway, however, is fecal excretion.

Chittenden et al. reported a mean absorbed dose coefficient to the bone surfaces of about 5 Gy/MBq and to the red bone marrow of 0.4 Gy/MBq (12).

Yoshida et al. provided mean absorbed doses for six Japanese patients (13). As a result, the authors observed mean absorbed dose coefficients in osteogenic cells of 0.76 Gy/MBq and 0.09 Gy/MBq in the red bone marrow (13).

Pacilio et al. (15) reported, in an Italian multicenter trial in which the dosimetry was based on quantitative imaging, that the mean effective half-life [^223^Ra]RaCl_2_ in bone lesions is 8.2 d and the absorbed dose after the first injection was 0.7 Gy (range 0.2–1.9 Gy).

Another model-based dosimetry calculation was published by Höllriegl et al. (16) who adopted the newest model of the ICRP [ICRP 137, (17)]. For most organs, their results were in the same range as those reported by Lassmann and Nosske (10), except kidneys and endosteal cells. The absorbed dose coefficient for the liver (alpha contribution) reported by Lassmann and Nosske (10) is almost identical to that of Höllriegl et al. (16) (36 mGy/MBq vs. 34.4 mGy/MBq). However, Höllriegl et al. (16) cited the value by Lassmann and Nosske (10) too low by a factor of ten.

To compare the dosimetry data for both radionuclides the absorbed dose coefficients were taken from the tables provided by Lassmann et al. (8, 10). The data for blood were taken from Schumann et al. (14) and Stephan et al. (18).

### Comparison of absorbed doses to organs or tissues

In Table 1, the ratios of the absorbed dose coefficients ([^224^Ra]RaCl_2_ vs. [^223^Ra]RaCl_2_) and, for a comparative analysis, of the absorbed doses of two treatment scenarios (10 MBq [^224^Ra]RaCl_2_ vs. 25 MBq [^223^Ra]RaCl_2_, corresponding to 6 cycles of 55 kBq/kg for a 75kg patient) are shown. A direct comparison between the activities administered in the study published by Groth et al. (5) (mean value of the high activities of 25 MBq [^224^Ra]RaCl_2_) to a standard treatment with [^223^Ra]RaCl_2_ (25 MBq [^223^Ra]RaCl_2_) is provided by the direct comparison of the absorbed dose coefficients. The activities for the two treatment scenarios were chosen to reflect the [^224^Ra]RaCl_2_ administered activities in the “Wick Study” and the activity administered for a standard treatment with [^223^Ra]RaCl_2_ to a 75 kg patient.

**Table 1:** Ratio of the absorbed organ dose coefficients (mGy/MBq) and the absorbed doses for typical administrations (10 MBq [^224^Ra]RaCl_2_ in the “Spiess study”, 25 MBq [^223^Ra]RaCl_2_ for six cycles in a patient of 75 kg).

**Organ**	**Ratio of absorbed dose coefficients: [^224^Ra]RaCl_2_/[^223^Ra]RaCl_2_**	**Ratio of Absorbed doses: 10 MBq [^224^Ra]RaCl_2_/25 MBq [^223^Ra]RaCl_2_**
Adrenals	0.73	0.29
Bladder wall	0.70	0.28
Bone endosteum	0.58	0.23
Brain	0.71	0.28
Breast	0.70	0.28
**GI-tract**		
Esophagus	0.71	0.28
St wall	0.72	0.29
SI wall	0.78	0.31
ULI wall	0.46	0.18
LLI wall	0.57	0.23
Colon	0.54	0.22
Kidneys	2.27	0.91
Liver	0.37	0.15
Muscle	0.72	0.29
Ovaries	0.97	0.39
Pancreas	0.72	0.29
Red marrow	0.57	0.23
**Respiratory tract**		
ET airways	0.67	0.27
Lungs	0.67	0.27
Skin	0.71	0.28
Spleen	0.88	0.35
Testes	0.87	0.35
Thymus	0.71	0.28
Thyroid	0.71	0.28
Blood	1.57	0.66

The data are taken from Lassmann and Nosske (10) and from Lassmann et al. (8) as the unweighted sum over the alpha and beta radiation contributions for each organ. The data for blood were taken from Schumann et al. (14) and Stephan et al. (18).

For most organs or tissues all decay products contribute almost equally to the absorbed doses in these organs (14, 16). Experimental data on these effects, however, are sparse and are taken from animal experiments (19). For the red marrow as primary organ-at-risk, the ratio of the absorbed dose coefficients is 0.57. The largest dissimilarities of the absorbed dose coefficient ratios are observed for the kidneys (2.27), blood (1.67), and liver (0.37). The higher values for the kidneys and blood could be attributed to the accumulation of lead and its progeny due to the longer half-life of ^212^Pb compared to ^211^Pb.

A comparison of the absorbed dose ratios assessed for the two treatment scenarios shows that the absorbed doses are always lower for [^224^Ra]RaCl_2_. For obtaining equal absorbed doses to the red marrow, the administered activity for [^224^Ra]RaCl_2_ can be chosen to be approximately 1.8-fold higher than that for [^223^Ra]RaCl_2_.

This comparison does not include absorbed doses of metastases which take up radium as the underlying ICRP models do not consider this case as they were designed for radiation protection purposes. Therefore, the absorbed doses to organs/tissue could be much lower if a considerable amount of the injected activity is taken up by tumors as only a fraction of the remaining activity will be available and taken up by other organs or tissues.

## External exposure

To further elucidate potential differences between ^224^Ra and ^223^Ra and their respected progenies regarding exposure of staff or persons staying close to patients, the dose rate constants for the ambient dose H^*^ were compared. For the comparison, the newest published values were used for ^224^Ra and ^223^Ra and the respective progenies (20).

The values for both radionuclides and their decay products are quite similar [45.17 μSv m^2^/(h GBq), ^223^Ra and 49.44 μSv m^2^/(h GBq), ^224^Ra]. The dose rate by a patient after administration of ^223^Ra in 1 m distance immediately following administration is 0.05 μSv/(h MBq). This value is in good agreement with the mean values measured by Dauer et al. of 0.02 μSv/(h MBq) (21). Overall, the external exposure of both radionuclides is low compared to other treatments with radiopharmaceuticals.

## Long-term radiation-related effects

### Patient cohorts studying long-term radiation-related effects of [^224^Ra]RaCl_2_

There are two patient cohorts that were followed for long-term radiation-related effects after the use of [^224^Ra]RaCl_2_.

In several publications Nekolla et al. (3, 22, 23) followed the health of 899 persons that were included in the “Spiess study“. The mostly juvenile patients received, mainly between 1945 and 1955, multiple injections of [^224^Ra]RaCl_2_ (mean specific activity: 0.66 MBq/kg, corresponding to an injection of 46 MBq to a 70 kg patient) with the aim of treating tuberculosis (TB), AS and some other diseases.

A second patient cohort included 1,471 AS patients treated with repeated intravenous injections of 0.17 MBq/kg [^224^Ra]RaCl_2_ between 1948 and 1975 (2). These patients have been followed in the “Wick study” together with a control group of 1,324 AS patients treated neither with radioactive drugs nor with X-rays. The mean follow-up time was 26.3 years in the exposed and 24.6 years in the control group.

### Radiation-induced side-effects of [^223^Ra]RaCl_2_ and [^224^Ra]RaCl_2_

In the study cohort of the “Spiess study”, Nekolla et al. (22) and Nekolla et al. (3) observed shortly after [^224^Ra]RaCl_2_ injections an increase in bone tumor risk significantly greater for younger ages at exposure. Most of the malignant bone tumors were osteosarcomas and fibrous-histiocytic sarcomas. During the two most recent decades of observation, a significant excess of non-skeletal malignant diseases has also become evident. Until the end of 2007, the total number of observed malignant non-skeletal diseases was 270 compared to 192 expected cases (3).

For [^224^Ra]RaCl_2_ the most striking observation of the “Wick study” (2) were the 21 cases of leukemia in the exposed group (vs. 6.8 cases expected, *P* < 0.001) compared to 12 cases of leukemia in the control group (vs. 7.5 cases expected). This increase in total leukemias was significant in direct comparison between the exposed and control groups too (*P* < 0.05). Wick et al. found, besides an increased standardized incidence ratio (= ratio of the number of observed cases vs. the number of expected cases) of leukemias, a significant increase for kidney and thyroid cancer (2).

For [^223^Ra]RaCl_2_ only mild side and mostly transient effects were observed (1). [^223^Ra]RaCl_2_ was well tolerated by patients with skeletal metastases. Mild to moderate and transient hematological toxicity was observed at potentially therapeutic doses. Platelets were less affected than neutrophils and white blood cells; toxicity grade I was seen in 5 of the 31 patients (1). Furthermore, only two cases of leukemia have been reported until today (24).

## Discussion

A major drawback for image-based dosimetry of [^223^Ra]RaCl_2_ is the inherent difficulty to quantify post-therapeutic gamma camera images, although photon emissions suitable for gamma camera imaging are available at ~82 keV, ~154 keV, and ~270 keV. Due to the low photon abundance, the low activities administered to the patients, and the high contribution of down-scatter of higher energy photons leading to severe septal penetration causes large image quantification uncertainties as reported by Hindorf et al. (25). Pacilio et al. (26) and Yoshida et al. (13) provided quantitative results by planar imaging, however, the accuracy of the respective quantification process for *in-vivo* imaging is even more limited due to activity overlay in this type of image. For ^224^Ra, data on imaging, though theoretically possible with the 239–241 keV gamma rays for ^224^Ra and ^212^Pb, and the 73–87 keV gamma rays of ^212^Pb and ^208^Tl have not been published. A feasibility study on how to quantify the decay product ^212^Pb by SPECT/CT imaging was published by Kvassheim et al. (27). However, the direct comparability of the results of this phantom study with activities up to 8 MBq to patient studies with [^224^Ra]RaCl_2_ is limited. For example, the [^224^Ra]RaCl_2_ activities administered in the patient study of Groth et al. (5) (maximum 30 MBq over several cycles) were at least one order of magnitude lower as compared to a recent clinical study with ^212^Pb-DOTAMTATE (28) (188 MBq per cycle for a 75 kg patient), thus hampering reliable image quantification of [^224^Ra]RaCl_2_.

A major concern for the application of radium isotopes to patients could be diffusion of the first daughter products ^219^Rn (half-life: 4 s) or ^220^Rn (half-life: 56 s). This could lead either to an increased diffusion of radon away from the binding site leading to unwanted irradiation of other organs or tissues or to increased emanation of radon, therefore reducing the energy deposited in the tumor/lesion.

Lloyd et al. (29) studied the retention, distribution and dosimetry of injected [^224^Ra]RaCl_2_ in six young adult beagles which were killed 0.04 to 7 days after [^224^Ra]RaCl_2_ administration. Their results suggest that, for the beagles, a fraction of roughly 0.08 of ^220^Rn or ^216^Po is produced *in vivo* and escapes from the skeleton. Increased *in-vivo* emanation of ^220^Rn was not observed in a study by Klemm et al. (30) who were looking for increased ^220^Rn exhalation in two AS patients after therapy with [^224^Ra]RaCl_2_.

Why it might be more favorable to use [^224^Ra]RaCl_2_ as compared to [^223^Ra]RaCl_2_ to treat solid tumors is shown in two studies by Arazi et al. (31) and Arazi (32). Although a different set-up - diffusing alpha-emitters radiation therapy utilizing implantable sources carrying small activities of ^224^Ra - the arguments are applicable also to the case of bone metastases taking up ^224^Ra. The released atoms disperse inside the tumor by diffusive and convective processes, creating, through their alpha emissions, a high-dose region measuring several millimeter in diameter about each source. If the decay point of ^220^Rn is effectively the starting point for the migration of ^212^Pb which may further distribute away from the source, the assessment by Arazi et al. (31) and Arazi (32) demonstrates that the size of the region subject to alpha particle irradiation may be expected to be of the order of millimeters rather than a few dozen micrometers. This might lead to a more homogeneous dose distribution in the tumor as compared to ^223^Ra. Similar findings have been reported by Napoli et al. in an experimental study with ^224^Ra-labeled CaCO_3_ microparticles (33). These considerations are not taken into account in any of the absorbed dose calculations until today (8, 10, 16).

Data on the biological effects by [^223^Ra]RaCl_2_ or [^224^Ra]RaCl_2_ are sparse. For ^224^Ra]RaCl_2_, only the publication by Stephan et al. (18) showed radiation dose-related effects on chromosomal aberrations in peripheral lymphocytes after repeated treatments. The frequency of chromosomal aberrations observed during the course of therapy was related to the absorbed dose to the blood. They also observed, that the frequency of dicentric chromosomes induced *in vivo* agreed well with the corresponding value of dicentrics induced *in vitro* (18).

For [^223^Ra]RaCl_2_ Sciuto et al. showed high dose dependent increase of the number of dicentrics and micronuclei during the course of [^223^Ra]RaCl_2_ therapy. The authors found a linear correlation between the absorbed dose to the blood and the number of dicentrics after repeated treatments.

Our group could show in several publications in peripheral blood mononuclear cells (PBMCs), by using the γ-H2AX assay as a marker for DNA double strand breaks, that there is, after internal *ex vivo* irradiation, a linear correlation between the number of alpha tracks induced by [^223^Ra]RaCl_2_ and [^224^Ra]RaCl_2_ revealing no difference between the radionuclides at the same absorbed dose (6, 34). Furthermore the *ex vivo* repair kinetics of the DNA damage in PBMCs is similar to the repair rate when compared to beta irradiation (35). Schumann et al. also observed *in vivo* in 9 patients after treatment with [^223^Ra]RaCl_2_ that the DNA damage is partly repaired (14).

Concerning long-term side effects, Priest et al. (36) compared, in a reanalysis of the AS patient data of the Wick study, the higher incidence of radiation-induced cancer with the fact that the patient treatment resulted decreased pain and increased mobility. Both of which are associated with decreased mortality by non-cancer diseases and from all causes of death. In their analysis they found no excess mortality in the group of AS patients. According to the authors, “the study demonstrates the need to consider all causes of death and longevity when assessing health impacts following irradiation” (36).

With respect to long-term effects of treatment with [^223^Ra]RaCl_2_, stochastic radiation-induced side-effects, although observed for [^224^Ra]RaCl_2_, are less relevant in the context of cancer treatment of prostate cancer as the median survival time of patients after treatment is 14 months (1). This is significantly less than 2 years considered to be the latent period for induced leukemia or the 8 year average latent period for induced bone cancer (23, 37, 38). Therefore, presently the benefit of the treatment of prostate cancer patients with [^223^Ra]RaCl_2_ outweighs the hypothetical risk associated with this treatment.

## Conclusions

When comparing the dosimetry data obtained by model-based calculations on [^223^Ra]RaCl_2_ and [^224^Ra]RaCl_2_ or data obtained by bio-dosimetric methods no major differences are observed for most organs. For kidneys, liver and blood the differences, most likely, can be explained by the differing half-lives of the respective progenies. Due to the difficulties associated with quantitative imaging of radium isotopes, absorbed doses derived by imaging procedures are less reliable due to inherent difficulties of image quantification. Furthermore, *in vivo* diffusion by radium progeny particularly in tumors is not well characterized and might need further experimental verification.

Data on long-term radiation-associated side effects are only available for treatment with [^224^Ra]RaCl_2_. In several studies, an increased cancer mortality by leukemia and solid cancers was observed. Similar considerations likely apply to [^223^Ra]RaCl_2_ as the biokinetics and the absorbed doses are in the same range, but this increased risk may not be observed due to the much shorter life expectancy of prostate cancer patients treated with [^223^Ra]RaCl_2_.

## Author contributions

Both authors listed have made a substantial, direct, and intellectual contribution to the work and approved it for publication.
